# Studies on Upgradation of Waste Fish Oil to Lipid-Rich Yeast Biomass in *Yarrowia lipolytica* Batch Cultures

**DOI:** 10.3390/foods10020436

**Published:** 2021-02-17

**Authors:** Agata Urszula Fabiszewska, Bartłomiej Zieniuk, Mariola Kozłowska, Patrycja Maria Mazurczak-Zieniuk, Małgorzata Wołoszynowska, Paulina Misiukiewicz-Stępień, Dorota Nowak

**Affiliations:** 1 Department of Chemistry, Institute of Food Sciences, Warsaw University of Life Sciences-SGGW, 159c Nowoursynowska Street, 02-776 Warsaw, Poland; bartlomiej_zieniuk@sggw.edu.pl (B.Z.); mariola_kozlowska@sggw.edu.pl (M.K.); patrycja.mazurczak@op.pl (P.M.M.-Z.); 2 Łukasiewicz Research Network—Institute of Industrial Organic Chemistry, 6 Annopol Street, 03-236 Warsaw, Poland; malgorzata.woloszynowska@ipo.lukasiewicz.gov.pl; 3 Postgraduate School of Molecular Medicine, Medical University of Warsaw, 2a Trojdena Street, 02-091 Warsaw, Poland; pmisiukiewicz@wum.edu.pl; 4 Department of Food Engineering and Process Management, Institute of Food Sciences, Warsaw University of Life Sciences-SGGW, Nowoursynowska Street 159c, 02-776 Warsaw, Poland; dorota_nowak@sggw.edu.pl

**Keywords:** oxidative stability, polyunsaturated fatty acids, single cell oil, waste fish oil, *Yarrowia lipolytica*

## Abstract

The aim of the study was to evaluate the possibility to utilize a fish waste oil issued from the industrial smoking process in nitrogen-limited *Yarrowia lipolytica* yeast batch cultures. The waste carbon source was utilized by the yeast and stimulated the single cell oil production via an ex novo pathway. The yeast biomass contained lipids up to 0.227 g/g d.m.. Independently from culture conditions, high contents of very long chain fatty acids were quantified in yeast biomass including docosahexaenoic (DHA), eicosapentaenoic acid (EPA), eicosenic and erucic acids. The pH regulation did not influence the cellular lipids yield (0.234 g/g d.m.). Meanwhile, the intensification of the oxygenation of medium by changing the mixing speed (maximum concentration of lipids produced 4.64 g/dm^3^) and decreasing the amount of inoculum had a positive effect on the culture parameters in waste fish oil medium. Further work on upgradation of the original waste is advisable, especially because the oil indicated high content of polyphenols and lower susceptibility to oxidation than microbial oil derived from control olive oil medium.

## 1. Introduction

Fish consumption and seafood popularity have been growing because of the undeniably benefit to human health. Therefore, fish wastes are predicted to arise worldwide and they stand for one of the continuously gaining grounds for waste management fields [1,2]. In recent years, there has been a constant increase in the exploitation of fish resources and the estimated quantity used for human consumption is 105.6 million tons (75% of the worldwide fish production). The remaining 25% (34.8 million tons) are considered as wastes. Solid waste represents 20–60% of the initial raw material [3]. Two conventional methods of waste utilization have been used nowadays. The wastes are being reused as either animal feed (composting, ensilation and fermentation of waste) or as a fertilizer [2]. Interestingly, bioremediation and biostimulation are biotechnological strategies that eliminate environmental pollutants along with growth stimulation of indigenous microorganisms [4].

A prominent approach to upgrade some fishery industry wastes involves their biotechnological reprocessing. The recovery of chemical components from seafood waste material (e.g., enzymes, gelatin, chitosan, chondroitin sulfate and proteins) is a promising area of research and development for the utilization of fish by-products. Enzymes and bioactive peptides obtained from fish waste may be used for fish silage, fish feed, source of gelatin or fish sauce production as a source of flavoring compounds, e.g., shrimp sauce [2,3,5,6]. Proteolytic hydrolyses were reported to produce peptides with interesting biological activities (anti-hypertensers, immune-modulators, antioxidants, anticoagulants, etc.) useful in the treatment of several diseases [7]. Lastly, fish oil extracted from marine fish was a source of omega-3 polyunsaturated fatty acids [8]. 

*Yarrowia lipolytica* is an intensively studied model oleaginous fungus that degrades efficiently hydrophobic substrates including lipids and carbohydrates, producing organic acids, aroma compounds, erythritol, enzymes, single cell protein (SCP) and single cell oil (SCO) [9,10]. Moreover, the ability of the yeast to form biofilms, encounters the chance of survival for cells during bioremediation and waste treatment studies [11,12]. Thus, many environmental and industrial applications of the microorganism were reported. Different strains of *Y. lipolytica* have been used for the treatment or upgrading of a variety of wastes; in particular, they were effective in the treatment of olive mill wastewater (OMW) and palm oil mill effluents (POME). Using inexpensive medium in microorganism culture is one of methods of reducing the costs of microbial metabolites production. This approach offers a two-fold advantage—a waste disposal and the synthesis of value-added products—and can be a commercially viable process [13].

The aim of the study was to evaluate whether the yeast strain *Y. lipolytica* KKP 379 is able to utilize a fish waste oil issued from the industrial smoking process. The target of the present investigations was to determine yeast growth and lipid accumulation when fish waste oil was used as a carbon source in nitrogen-limited batch cultures. The data obtained were analyzed to study the changes in fatty acid profile among initial carbon source, cellular lipids and residual oil remaining in the growth medium. Moreover, the influence of pH and aeration on yeast biomass yield and lipid synthesis was described. The future perspective of the presented solution was also outlined and some features of the extracted microbial oil were evaluated.

## 2. Materials and Methods

### 2.1. Microorganism

*Y. lipolytica* strain KKP 379 from the Collection of Industrial Microorganisms at the Prof. Wacław Dąbrowski Institute of Agricultural and Food Biotechnology in Warsaw (Poland) was used in the study. The strain has been stored in cryovials containing ceramic beads with a cryoprotective agent at −20 °C (Protect Select, Technical Service Consultants Ltd., Heywood, UK).

### 2.2. Waste Fish Oil

Waste fish oil was used as a carbon source in *Y. lipolytica* culture medium. The oil was collected in tanks after the smoking process from the fish factory located in the Podlaskie Voivodeship in Poland. The company produces the following fish products: smoked, fried and fresh fish, and the smoking process is carried out mainly on salmonids (*Salmonidae*) and mackerels (*Scombridae*). Full characteristic of the waste oil was presented in Table 1.

### 2.3. Media and Culture Conditions

Inoculum was prepared in YPG medium with the following composition: 10 g/dm^3^ yeast extract (Y), 20 g/dm^3^ peptone (P), 20 g/dm^3^ glucose (G); pH = 5. The flasks were incubated at 140 rpm, 28 °C for 24 h. Number of yeast cells in inoculum pre-culture was determined by plate method on YPG agar medium and it ranged from 8.0 to 8.5 log CFU/cm^3^ (average 8.29 ± 0.21 log CFU/cm^3^). All medium ingredients were purchased from BTL (Łódź, Poland).

All fermentation experiments were conducted with 50 g/dm^3^ waste fish oil and C:N ratio of 83:1 mol/mol (MF5 medium). Control medium contained 50 g/dm^3^ olive oil (Aceites Borges Pont, Spain) and C:N ratio of 85:1 mol/mol (MO5 medium). The other medium components were, g/dm^3^: KH_2_PO_4_, 7; (NH_4_)_2_SO_4_, 2.5; Na_2_HPO_4_, 2.5; FeSO_4_ × H_2_O, 0.16; CaCl_2_, 0.15; MnCl_2_ × 4H_2_O, 0.08; ZnSO_4_, 0.02; yeast extract, 2.0; peptone, 1.0. All inorganic salts were purchased from Avantor Performance Materials Poland S.A (Gliwice, Poland). The initial pH of medium was estimated at 6.0 based on the preliminary studies (data not shown). Yeast were cultivated at 28 °C, with a rotation of 350 rpm in a BioFlo 3000 bioreactor (New Brunswick Scientific, Germany). To determine pH of culture, the pH glass electrode was used. Medium was aerated with compressed air at a flow of 105 dm^3^/h per 1 dm^3^ medium. Inoculum for the bioreactor was standardized by measuring the optical density of the culture and amounted 0.0025% *v/v* or 0.0075% *v*/*v*. During batch culture with regulated aeration variable speed of the agitator changed from 350 to 600 rpm (every 50 rpm), so that the oxygen level remained within 20% of the initial oxygenation of the medium. The growth phases of yeast cells were determined on the basis of the oxygenation of the medium, which was expressed as a relative percentage of dissolved oxygen in the medium in relation to its concentration at the beginning of the culture (dissolved oxygen, dO2), measured by using of an oxygen electrode. During batch culture with regulated pH the parameter maintained constant at the level of 5 by addition of 25% ammonia solution. The batch cultures were performed twice.

### 2.4. Determination of Biomass

Yeast biomass was characterized by cell dry mass measured by the thermogravimetric method. Cells were harvested by centrifugation (8000 rpm, 10 min, 4 °C), washed in distilled water and dried at 105 °C until constant weight. 

### 2.5. Cellular Lipids and Residual Oil Extraction and Analysis

Cellular lipids were extracted and analyzed by gas chromatography method according to Fabiszewska et al. [14]. A total of 20 to 40 g of wet biomass was harvested by centrifugation (8000 rpm, 10 min, 4 °C) for lipid extraction, washed with distilled water and dried at 80 °C. Extraction was performed in Soxhlet extractor using *n*-hexane as a solvent. The residual content of lipid source was determined by twice lipid extraction from 200 cm^3^ of medium with *n*-hexane and measuring of oil weight after solvent evaporation. 

### 2.6. Determination of Total Phenolic Content

The content of phenolics in studied samples was determined using the Folin–Ciocalteu’s reagent [16] with some modification described by Krzyczkowska and Kozłowska [17]. Briefly, 1 g of oil sample was dissolved in 5 mL of *n*-hexane, and 5 mL of methanol (80%) was added. The mixture was centrifuged and the methanolic layer was separated from the lipid phase. Then, 0.5 mL of the methanolic layer was diluted in water and then the Folin–Ciocalteu’s reagent (0.5 mL) and 1 mL of a sodium carbonate solution (20%) were added. The absorbance was measured at 760 nm after 60 min with the samples standing in the dark. The total phenolics content in each oil sample was determined using a standard curve plotted for gallic acid. The results were expressed as mg gallic acid per gram of oil. 

### 2.7. DSC Measurements

The Q20P pressure differential scanning calorimeter (TA Instruments, New Castle, Delaware USA) with high pressure DSC cell (Q Series DSC Pressure Cell, PDSC) was used, but the experiments were performed under normal (atmospheric) pressure of oxygen gas flowing at a rate of 6 L/h. The instrument was calibrated using high-purity indium metal standard. Oil samples 3.5 ± 0.5 mg were placed in aluminum sample and were heated at a constant heating rates (*β*) of 4–15 °C min^−1^. For each experiment and each programmed heating rates at least triplicate determinations were carried out. From the resulting oxidation exotherms the oxidation onset temperatures (t_ON_, °C) were determined as the intersection of extrapolated baseline and the tangent line (leading edge). Determination of kinetic parameters was the same as described in the previous papers [18,19].

### 2.8. Statistical Analysis

Statistical analyses were performed of repeated measurements with the one-way ANOVA followed by Tukey’s multiple comparison test and analysis of correlation using STATISTICA 13.1 (Statsoft, Poland). *p*-values lower than 0.05 were considered to be statistically significant. The Shapiro–Wilk test was used to check if the populations were normally distributed, while Levene’s test and the Brown–Forsythe test were used to assess the equality of variances for a variable calculated for groups.

## 3. Results

### 3.1. Single Cell Oil Synthesis in Batch Culture of Y. lipolytica

**Figure 1:**
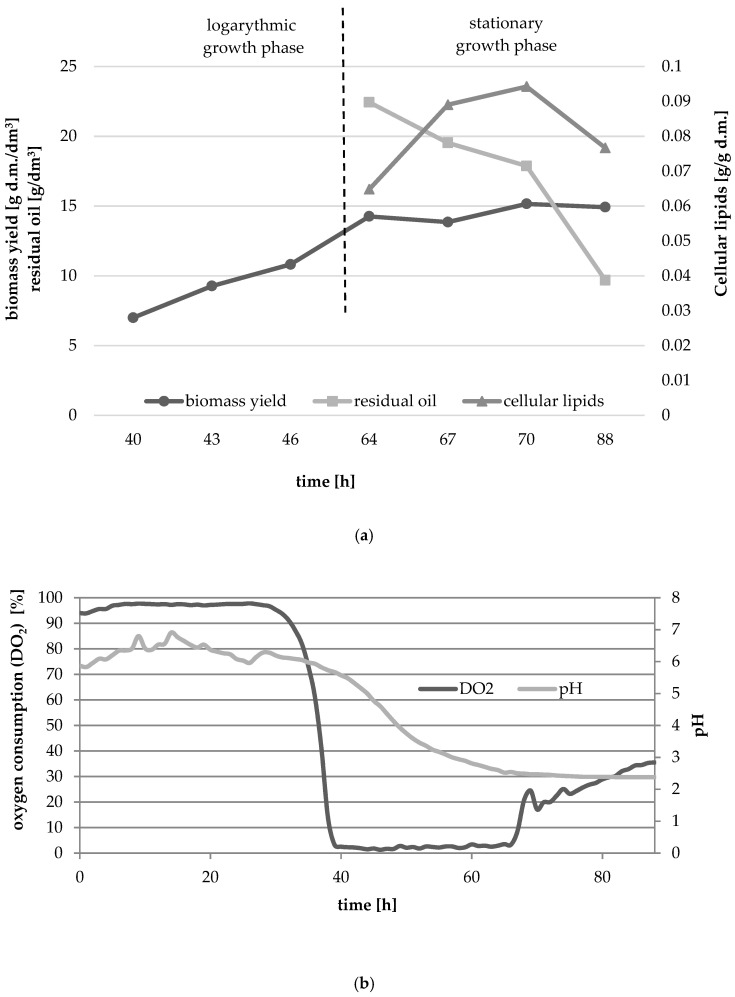
Changes in biomass yield and the content of cellular lipids (**a**), relative oxygen consumption and pH (**b**) of the yeast strain *Y. lipolytica* KKP 379 grown in MF5 medium with waste fish oil as a carbon source.

The main purpose of the experiment was to attempt the ex novo synthesis of single cell oil in a medium containing waste fish oil in *Y. lipolytica* batch culture. Biomass yield and content of microbial oil in yeast were determined in biomass collected between 64 and 88 h of culture which corresponded to late logarithmic and early stationary growth phases. Figure 1a,b show the growth of yeast culture in medium with waste fish oil as a carbon source. The culture was characterized by a very long adaptation phase (about 37 h), and dry biomass yield in 40 h of culture, i.e., in the initial logarithmic phase was 7 g d.m./dm^3^. During the experiment biomass yield increased, reaching the highest value in 70 h of culture (15.16 g d.m./dm^3^). The content of microbial oil also changed over time (Figure 1a). After 64 h the lipid yield amounted 0.065 g/g d.m. and after 70 h of culture the highest lipid yield was measured (0.094 g/g d.m.). Extending the culture to 88 h caused a slight decrease in the dry biomass yield (14.92 g d.m./dm^3^) and in the cellular lipids content (0.077 g/g d.m.). The decrease in the content of microbial oil in cells may be caused by its partial consumption and use for energy purposes by β-oxidation of fatty acids [20]. At the beginning of stationary growth phase there was observed almost half of the carbon source added at a culture starting point (Figure 1). Even though, the biomass yield did not change significantly in the growth phase, waste fish oil was still being consumed for SCO synthesis. 

It should be pointed out that the long lag phase was expected as the extracellular lipase activity was low in the first two days of culture (data not shown). Comparing the lipase profile of cells in MO5 medium, the highest lipase activity was observed during early logarithmic phase. The lipolytic enzyme is needed for triacylglycerols hydrolysis and required for free fatty acids assimilation. Following the literature data, the oleic acid is considered as the best fatty acid stimulating *LIP2* gene promotor [9]. The FA profile of waste oil an olive oil are far from identical and the content of C18:1 fatty acid is different (Table 2, oleic acid content is 17.30 and 76.10, respectively). This appears to be one of the factors stimulating the lipolytic activity of the yeast cells.

The pH decreased in the logarithmic and stationary phases (Figure 1b) probably as the citric acid and other carboxylic acids were being secreted to the medium and were synthesized alongside with the depletion of nitrogen source and disruption of Krebs cycle. The minimum level of the pH was 2.5.

The fatty acid profiles of microbial oils (Table 2) obtained at two different stages of the experiment in MO5 medium containing olive oil, i.e., from 26 (logarithmic phase) and 38 h of culture (stationary phase, data not shown), were compared. The time of sampling was chosen so as to characterize the similar phase of growth with MF5 culture. Olive oil is a commonly used hydrophobic substrate in lipolytic microorganisms culture, so the cells from MO5 medium were the reference. There was also a slight decrease in the content of polyunsaturated fatty acids (linoleic acid) compared to its content in olive oil. Lengthening of the culture led to changes in the content of palmitic and oleic acids. Sixteen-carbon saturated palmitic acid, similarly as in the case of culture in a medium with waste fish oil, could be used for cell growth, and an increase in oleic acid content may indicate that it is the main component of the yeast storage lipids. In microbial oil extracted from yeast cells grown in MO5 medium, the content of unsaturated oleic acid decreased from 76 to about 50%, and the content of saturated acids, especially palmitic acid, increased to a value exceeding 20% of all fatty acids. Microbial oil which was extracted from cells grown in a medium with waste fish oil contained omega-3 polyunsaturated fatty acids, i.e., EPA and DHA (Table 2). Their content in cellular lipids was comparable to the carbon source (respectively 8.0% and 10.60% for waste oil and 7.26% and 9.97% for microbial oil collected in 88 h of culture). What is not obvious was that in the MO5 medium, cells accumulated the fatty acids of 20 or more carbon atoms in the fatty acid residue (behenic acid, erucic acid and lignoceric acid). Similarly, there were detected arachidic, behenic, lignoceric and nervonic fatty acids in SCO extracted from MF5 grown *Y. lipolytica* cells.

### 3.2. Impact of Culture Conditions on SCO Synthesis in Waste Fish Oil Media

There was evaluated the impact of aeration and pH regulation on the course of yeast growth and storage lipids yield in waste fish oil medium (MF5). The control media contained olive oil or glucose as the carbon source (MG5 and MO5) (Table 3). The intensification in the oxygenation of medium by changing the mixing speed (20% of air saturation, agitation rate 400 rpm) resulted in a significant increase in biomass yield up to 20.43 g d.m./dm^3^. Compared to cultivation without regulation of the parameter the biomass yield was 15.16 g d.m./dm^3^. On the other hand, the regulation of pH on the constant level did not influence the yeast growth positively, but both growth condition changes (pH regulation and higher oxygenation) resulted in an increase in the maximum concentration of lipids produced (L) compared to the SCO yield obtained in the MF5 batch culture. The L_max_ value was 1.430 g/dm^3^, respectively, in unregulated culture, 1.983 g/dm^3^ using pH regulation and 4.638 g/dm^3^ in the oxygenated culture. The highest values of volumetric rate of storage lipids production (q_Ly_) and the average volumetric specific rate of storage lipid production (qL) were recorded in culture with controlled medium oxygenation and they were more than twice higher than in the case of culture in the same medium, but without regulation of the oxygen concentration in medium. The maximum concentration of lipids produced in control MG7 medium was very low in comparison to MO5 medium (1.15 and 7.88 g/dm^3^, respectively), which made olive oil the most highly converted carbon source into SCO (0.340 g/g d.m.).

**Figure 2:**
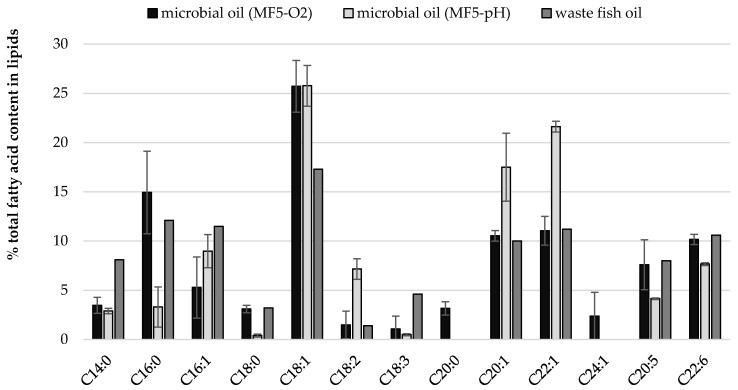
Comparison of fatty acid content in waste fish oil (carbon source in growth medium) and cellular lipids from yeast cell grown in MF5 medium in cultures where aeration (MF5-O_2_) and pH (MF5-pH) parameters were modified.

**Figure 3:**
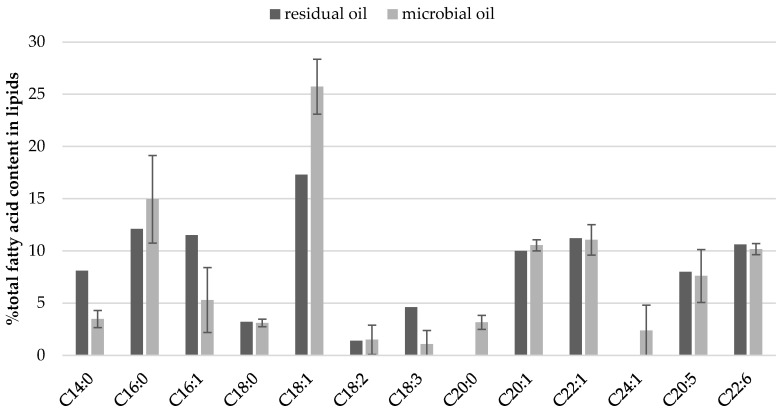
Parallel of fatty acid content in cellular lipids synthesized in MF5-O_2_ medium and in residual oil in supernatant at the end of yeast culture in stationary growth phase.

The impact of culture conditions such as pH and oxygen regulation on fatty acid profile of cellular lipids (Figure 2 and Figure 3) was investigated. Some general regularities were observed like twice lower content of C14:0 in SCO than in fish oil, but 10% lower content of oleic acid (C18:1). Once again, independently from culture conditions, high contents of very long chain fatty acids (VLCFA) were quantified in yeast biomass including EPA, DHA, eicosenic and erucic acids (Figure 2). Moreover, the yeast cells accumulated proportionally FFA from carbon source and slight differences can be observed for their content in storage lipids and waste fish oil medium (Figure 3). What is characteristic, apart from culture conditions, the fatty acids profile in lipids extracted from *Y. lipolytica* cells grown in oxygenated waste fish oil medium (MF5-O_2_) and cultured with regulated pH (MF5-pH) reflected the profile of FFA in waste oil (Figure 2, Table S1). 

The results of the fatty acid profile synthesized by the tested yeast strain on a synthetic medium with waste fish oil with those synthesized by cells on glucose medium (MG7) were compared (Table 2). Under these conditions, de novo synthesis took place. It is notable that under these conditions cellular lipids contained 56.37% of oleic acid, 18.44% of linoleic acid and no PUFA had been detected. A similar pattern of results was obtained in cellular lipids extracted from initial cells grown in YPG medium and used as inoculum for other cultures. Cells were rich in oleic acid (73.96%) and once more the PUFA were not detected.

**Figure 4:**
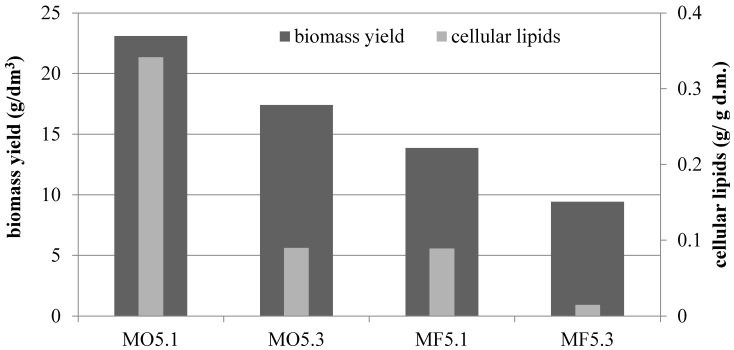
Comparison on biomass yield and cellular lipids amount isolated from yeast biomass cultured in batch bioreactor differing in carbon source in medium (olive oil—MO5 or waste fish oil—MF5) and amount of inoculum (0.0025% *v*/*v*—1; 0.0075% *v*/*v*—2).

Decreasing the amount of inoculum had a positive effect on the culture parameters in MO5 and MF5 media. In the olive oil medium, the content of cellular lipids reached the value of 0.341 g/g d.m. (when the lower volume of inoculum culture was used—0.025% *v*/*v*) and this result was almost four times higher than the one obtained for culture inoculated with 0.075% *v/v* (0.090 g/g d.m.) (Figure 4). When using waste fish oil as a sole carbon source in culture, this difference was even greater, because a 6-fold increase in the amount of accumulated lipids was observed (0.089 g/g d.m.). In the waste fish oil medium the trend was similar and 13.85 and 9.43 g d.m./dm^3^ were obtained, respectively.

### 3.3. Selected Quality Aspects of Microbial Oil of Y. lipolytica Obtained in Media with Waste Fish Oil

Microbial oils are regarded as promising alternatives to plant oils as concerns over environmental issues and a need for alternative food sources continue to arise [20]. As a consequence, a deep analysis of toxicity and storage quality should be undertaken in order to pose a valuable safe food and feed product. In the study, three main properties of the obtained microbial oil have been considered: the fatty acids profile (discussed in the previous chapters), oxidative stability and polyphenols content.

Table S2 presents the oxidation onset temperatures (t_ON_) of the microbial oil samples extracted from yeast cells grown in MO5 and MF5 media studied at varying heating rates (*β*). The t_ON_ is the parameter that is closely associated with the formation of hydroperoxides which may be rapidly decomposed to form a variety of secondary products, such as aldehydes, ketones, alcohols, hydrocarbons and polymers. These compounds are mainly responsible for sensory changes in food products and often lead to their deterioration. In our study, it was observed that the increase in the heating rate from 4 to 15 °C min^−1^ led to an increase in t_ON_ values for the tested oil samples. 

The kinetic parameters, namely activation energy, pre-exponential factor and induction time, which were calculated using t_ON_ values for the tested oil samples, are presented in Table S3. The activation energy (E_a_) values obtained for the oxidation reaction for MF5 and MO5 medium derived oil samples were 87.21 and 90.33 kJ mol^−1^, respectively. Considering also this parameter, which represents the minimum energy required for a reaction to start, it can be stated that oxidative stability of microbial oil obtained from cells grown in waste fish oil medium was higher than for olive oil medium. Based on another indicator for determining the stability of oils using DSC such as the induction time (τ values) calculated at 140 °C and 150 °C, it was also observed that the thermooxidative stability of oil samples studied was higher for SCO extracted from olive oil grown cells. 

As the presence of tocopherols and polyphenols in oil and the interactions between them can affect their oxidative stability, the total phenolic compounds content was determined (Table S4), which amounted to 1.22 mg GA/g oil in microbial oils extracted from yeast cells cultured in waste fish oil (MF5) and 0.035 mg GA/g in olive oil medium (MO5).

## 4. Discussion

### 4.1. Impact of Selected Factors Influencing Single Cell Oil Yield and the Fatty Acid Content 

According to Papanikolau and Aggelis [20], the oleaginous yeast species are capable of lipid synthesis using both biosynthesis mechanisms de novo and ex novo. De novo accumulation of cellular lipids involves citric and isocitric acids accumulation as the tricarboxylic acids cycle is blocked. Meanwhile, Fabiszewska et al. [21] indicated that de novo lipid biosynthesis could occur despite the presence of fatty acids in the medium, and the synthesis of storage lipids in the presence of lipid carbon sources could be carried out with the use of both pathways in *Y. lipolytica* cells (de novo and ex novo). In the present study, the low pH of supernatant and the composition of microbial oil might have acknowledged the hypothesis. 

In the studies of Saygun et al. [22], the yeast strain *Y. lipolytica* accumulated lipids in a medium with rapeseed oil, which is similar in composition to olive oil, and in both fats, the main fatty acid was oleic acid. According to the results of cited researchers, the content of oleic acid decreased from 62.8 to 49.9%, and palmitic acid increased from 5.5 to 18.8% relative to the content of all fatty acids [22]. Microbial oil analyzed by the authors was characterized by fatty acids profile similar to the carbon source used in medium. The present study confirms that in lipid-rich medium ex novo synthesis of lipids occur and most of the fatty acids are incorporated into the storage lipids in a non-modified form in *Y. lipolytica* cells.

There is a common certitude that the hydrophobic compounds could be both selectively taken from the medium, as well as partially metabolized and transformed as a result of the β-oxidation process. That would explain the change in the SFA, MUFA and PUFA proportions in lipids extracted from yeast cells cultivated in olive oil medium. Lipids derived from waste fish oil medium were more diverse according to fatty acids profile than in the olive oil medium but the SFA/MUFA/PUFA ratio did not change significantly in SCOs. Most likely, EPA and DHA long chain fatty acids were more slowly absorbed by yeast cells compared to other acids, which is why their share in the sum of fatty acids increased only at later stages of culture. Acids with shorter carbon chains, such as myristic (C14: 0), were taken up faster than other acids and used for growth, which is also confirmed in their research by Papanikolaou et al. [23]. Saygun et al. [22] evaluated the possibility of growth and production of metabolites such as citric acid and microbial oil by the yeast strain *Y. lipolytica* YB 423-12 in media containing vegetable oils, trout oil or pomace from vegetable oil production. The yeast strain accumulated only part of the EPA and DHA acids from the trout oil used in medium, due to which the PUFA content decreased approximately twice compared to the original fish oil.

Guo et al. [24] screened for yeast strains incorporating the exogenous EPA and DHA acids from crude fish oil, which were expected to be excellent sources for hen, fish and other animal feeds. *Y. lipolytica* FO726A was found to be the most efficient strain with a high yield of cell mass rich in EPA and DHA. The strain FO726A was determined to be unable to synthesize those fatty acids, which suggested that all EPA and DHA accumulated in cells come from the fish oil added [24]. Moreover, genetic manipulations allow the yeast to acquire the ability to synthesize polyunsaturated fatty acids along the de novo pathway. Using the modified strain of the *Y. lipolytica* species a DuPont dietary supplement called NewHarvest ™ was produced for few years. This supplement sold in capsule form contained oil with a significant amount of eicosapentaenoic acid (EPA). *Y. lipolytica* yeast biomass with a high EPA content is also used in feeding Verlasso^®^ farmed salmon [25]. Nevertheless, non-modified microbial sources of EPA and DHA are investigated and desirable.

There are some microorganisms such as *Schizochytrium* sp. that produce very long chain fatty acids (VLCFAs). Yeast *Y. lipolytica* is claimed not to able to synthesize longer than 18-carbon fatty acids due to the absence of many genes encoding elongases and desaturases responsible for elongation and formation of unsaturated bonds in fatty acid molecules. In the *Y. lipolytica* genome, genes of *Elo1* and *Elo2* elongases and delta-12-fatty-acid desaturases are found [26]. Nevertheless, *Yarrowia* cells accumulated the fatty acids of 20 or more carbon atoms in the fatty acid residue. This suggests that some modifications of incorporated lipid molecules can occur and those abilities could be strain-dependent. The above conclusions can be supported by Gajdos et al. [27] who reported the presence of some small amounts of VLCFA (less than 3% of the total fatty acid content) in *Y. lipolytica* W29 wild strain (arachidic, eicosenic, behenic and lignoceric acids).

The sufficient intensive mixing had an impact on increasing the cell division of *Y. lipolytica* KKP 379 yeast as the microorganism is obligately aerobic [28], for that reason the oxygenation of the medium increased the SCO yield. The obtained results indicated also that the use of a smaller amount of inoculum positively affected the growth of yeast cells, which is in contradiction with the results obtained by Rakicka et al. [29], who obtained a higher biomass yield using a higher density inoculum. The scientists determined the possibility of lipid synthesis by the genetically modified yeast strain *Y. lipolytica* JMY4086 in media containing molasses and waste glycerol. The experiments described by the authors showed that the content of intracellular lipids in the yeast cells was positively influenced by the use of an inoculum with a lower optical density and by not using oxygen regulation [29]. The results of the study presented by Taskin et al. [30] acknowledged that the amount of inoculum had a statistically significant effect on the growth and accumulation of lipids by the yeast strain *Y. lipolytica* B9. In the experiment, 6 variants of inoculum volume (from 1 cm^3^ to 6 cm^3^) with an optical density of 2 were used, and intracellular lipid content relative to dry matter ranged from 28% for the smallest inoculum volume to 20% for the largest volume. At the same time, the optimal value for lipid synthesis with a simultaneously high biomass yield was an inoculum volume of 3 cm^3^ [30]. Moreover, Liu et al. [31] utilized potato wastewater in culture of *Lipomyces starkeyi* GIM2.142. The selected sources of carbon and nitrogen, their concentration, culture time, as well as the size of the inoculum had a significant impact on cell growth and microbial oil accumulated inside the cell. Inoculum was tested in the range of 5–25%. In 96-h cultures the biomass yield increased with increasing inoculum, and the level of 10% of inoculum occurred the most beneficial in the case of lipid synthesis [31].

A reduced amount of inoculum, and therefore a higher ratio of initial yeast cell number respect to nutrients present in the culture medium may be the reason why yeast accumulate larger amounts of storage lipid. Nutrients could be consumed for energy and building processes, and the excess of collected components is stored in lipid bodies. 

### 4.2. Oxygen Stability in Single Cell Oil Influencing the Microbial Oil Usefulness

Kozłowska et al. [32,33] observed the increase in the oxidation onset temperature with increasing heating rate for the lipid fraction extracted from the cookies and sponge-fat cakes after baking and during their storage. In our study, the increase in t_ON_ values was higher when SCO from waste fish oil medium was studied. At the same heating rate, for example at 10 °C min^−1^, the t_ON_ values for both microbial oils were 137.53 and 192.94 °C min^−1^, respectively. Malvis et al. [34] also studied thermal oxidation stability of different varieties of olive oil using DSC at different heating rate 0.5–10 °C min^−1^. When comparing their results with our study, it was observed that for heating rate of 10 °C min^−1^, the three varieties of oils showed similar t_ON_ values. They were equal 190.0, 191.1 and 192.9 °C, respectively. 

The higher t_ON_ values obtained for microbial oil from MF5 medium may indicate a higher stability of this oil and thus its lower susceptibility to oxidation. This observation may be due to the fact that waste fish oil derived storage lipids contained lower amount of polyunsaturated fatty acids compared to oil from olive oil cultured yeast. The presence of a greater number of double bonds in the fatty acid chain promotes the production of more complex mixtures of hydroperoxides, which are easily decomposed and become very difficult to analyze quantitatively. The rate of oxidation depends on degree of unsaturation and increases with the increase of number of double bonds in fatty acids [34]. Tengku-Rozaina and Birch [35] reported that fish oil is susceptible to thermal oxidation due to their high PUFA content. They studied oxidative stability of hoki and tuna oils and showed that tuna oil was rapidly oxidized because it contained high percentage of PUFA, mainly DHA. Thermal decomposition of PUFA of tuna oil started at a lower temperature than in hoki oil and was also correlated with PUFA positional distribution in tuna oil triglycerides structure.

The presence of tocopherols and polyphenols in olive oil and the interactions between them can affect its oxidative stability. Although in our research it turned out that it probably not the polyphenols content determined the better resistance to SCO from MF5 cultured yeast cells oxidation compared to MO5 medium because the total phenolic compounds content was lower in cellular lipids from MF5 grown cells. In addition, Cerettani et al. [36] showed that increasing amounts of phenolic compounds do not seem to have influence on crystallization profiles or related thermal properties of unheated extra virgin olive oil under the experimental conditions applied. Similarly, Litwinienko et al. [37] showed that the use of the selected phenolic compound at a concentration greater than 10 mmol per mol of linolenic acid in the process of its thermooxidation studied by the non-isothermal DSC method was not the reason for their better antioxidant activity. On the basis of the presented study and comparison with the literature data, it can be concluded that the oxygen stability of microbial oil extracted from MF5 medium was good enough for food technology purposes. Nevertheless, the reasons for that remain unanswered fully and should be the scope of the future research.

Generally, the spectrophotometric and chromatographic techniques such as gas chromatography (GC), high performance liquid chromatography (HPLC) or capillary electrophoresis (CE) are utilized to identify and quantify individual phenolic compounds in the studied samples. Although, the most commonly used method for the determination of total phenolics content in samples is a colorimetric method based on electron transfer reaction between the Folin–Ciocalteu reagent and phenolic compounds. However, the Folin and Ciocalteu’s reagent does not react specifically with only phenols but also with other types of reducing substances that may be present in the studied sample and may influence the accuracy of the method and thus the content of phenolic compounds [38]. These compounds include reducing sugars (glucose and fructose), aromatic amines, ascorbic acid, dehydroascorbic acid (DHA), some organic acids and polyhydric alcohols. In addition, during processing of food containing phenolics, it is possible to convert them to a variety of derived compounds containing phenolic groups that oxidation of them may produce products which themselves become reducing agents thus giving a greater Folin value.

## 5. Conclusions

The nutritional value of the microbial oil obtained in *Y. lipolytica* yeast culture in waste fish oil medium resulting from the high content of unsaturated fatty acids including DHA and EPA and high polyphenols concentration, makes the lipid-rich yeast biomass a promising source of beneficious nutrients. Even though we did not explain fully the reasons for high stability of microbial oil derived from waste oil medium. It seems reasonable to continue the experiments on the upgradation of oily food-processing wastes in the *Y. lipolytica* yeast cultures also because of the fact that our results were broadly in line with global trends and sustainable development.

The limitations of the present studies naturally include also the maximum level of storage lipids accumulated in the yeast cell. However, when comparing our results obtained in waste fish oil medium to those of control olive oil medium, it must be pointed out that the SCO yield can be still improved by optimizing the culture conditions. Appropriate selection of the strain and culture conditions may result in obtaining valuable lipid-rich yeast biomass with simultaneous upgradation of lipid waste. The main factors determining strain selection are the ability to synthesize storage lipids in the minimum concentration of 20% in dry mass as well as the hydrophobic substrates utilization. Noteworthy, wastes are a mixture of different contaminants which can affect cell growth, so the biomass yield and growth curves should be carefully analyzed prior strain selection. The great advantage in developing the yeast strain might be genetic engineering, so the usage of model organism is beneficial. The usage of *Y. lipolytica* species gives an opportunity to improve the SCO oils and waste conversion in future research.

## Figures and Tables

**Table 1:** Chemical parameters of waste fish oil originated from fish smoking process.

Parameter	Unit	Method	Result
Lipid content	%	Extraction and weight method [14]	99.7 ± 0.2
Protein content	%	Kjeldahl method	<0.3
Carbohydrate content	%	Regulation (EU) No 1169/2011 of the European Parliament and of the Council of 25 October 2011 on the provision of food information to consumers	<0.1
Fiber content	%	Enzymatic-gravimetric method (AOAC 991.43:1994)	<0.5
Water content	%	Karl Fisher method (EN ISO 8534:2008)	0.23 ± 0.01
Energy value	kcal/100 g kJ/100 g	Regulation (EU) No 1169/2011 of the European Parliament and of the Council of 25 October 2011 on the provision of food information to consumers	897
			3689
Insoluble impurities content	%	Solvent extraction method (ISO 663:2017)	0.030 ± 0.001
Ash	%	Incineration and weight method (ISO 6884:1985)	0.01 ± 0.001
Sodium (Na)	g/100 g	Inductively coupled plasma mass spectrometry [15]	<0.01
Calcium (Ca)	mg/kg		<10
Magnesium (Mg)	mg/kg		<5
Phosphorus (P)	mg/kg		4.24 ± 0.03
Manganese (Mn)	mg/kg		<1
Arsenic (As)	mg/kg		<0.09
Lead (Pb)	mg/kg		<0.05
Mercury (Hg)	mg/kg		<0.0006
Cadmium (Cd)	mg/kg		<0.002

**Table 2:** Profile of fatty acids in carbon sources (waste fish oil, olive oil), initial yeast biomass grown in YPG medium and microbial oil extracted from yeast cells grown in MF5, MO5 and MG7 medium (content of fatty acids in relations to total fatty acids concentration, %).

Fatty Acid		Initial Yeast Biomass Grown in YPG Medium	Olive Oil (O)	Microbial Oil from *Y. lipolytica* Cells Grown in MO5 Medium		Waste Fish Oil (F)	Microbial Oil from *Y. lipolytica* Cells Grown in MF5 Medium			Microbial Oil from *Y. lipolytica* Cells Grown in MG5 Medium
Symbol	Name			26 h	38 h		64 h	70 h	88 h	
C14:0	Myristic acid	1.72 ± 0.07	−	−	−	8.10 ± 0.29	1.76 ± 0.09	2.25 ± 0.13	2.76 ± 0.17	−
C16:0	Palmitic acid	13.91 ± 0.29	9.30 ± 0.39	27.61 ± 0.51	22.70 ± 0.55	12.10 ± 0.34	13.06 ± 0.44	9.05 ± 0.32	8.96 ± 0.66	8.33 ± 0.40
C16:1	Palmitoleic acid	3.16 ± 0.53	−	−	−	11.50 ± 0.78	4.19 ± 0.67	3.50 ± 0.54	3.54 ± 0.45	5.27 ± 0.15
C18:0	Stearic acid	5.04 ± 0.10	3.10 ± 0.09	2.50 ± 0.07	2.54 ± 0.12	3.20 ± 0.15	1.31 ± 0.08	1.84 ± 0.06	1.45 ± 0.06	2.1 ± 0.11
C18:1	Oleic acid	73.96 ± 1.87	76.10 ± 1.87	51.46 ± 1.94	57.33 ± 1.98	17.30 ± 2.03	30.68 ± 1.67	29.20 ± 1.59	29.49 ± 1.47	56.37 ± 1.40
C18:2	Linoleic acid	0.76 ± 0.78	10.60 ± 0.51	7.74 ± 0.60	8.09 ± 0.89	1.40 ± 0.76	7.25 ± 0.86	5.76 ± 0.84	6.08 ± 0.96	18.44 ± 1.02
C18:3	Linolenic acid	1.45 ± 0.15	−	1.83 ± 0.09	1.37 ± 0.12	4.60 ± 0.21	1.76 ± 0.17	4.11 ± 0.23	1.50 ± 0.16	−
C20:0	Arachidic acid	−	−	−	−	−	4.45 ± 0.12	2.39 ± 0.15	2.83 ± 0.19	2.81 ± 0.25
C20:1	Eicosenic acid	−	−	−	−	10.00 ± 1.67	4.57 ± 1.21	5.82 ± 1.45	5.24 ± 1.34	1.81 ± 0.24
C22:0	Behenic acid	−	−	2.41 ± 0.10	1.75 ± 0.15	−	1.95 ± 0.19	2.96 ± 0.17	2.84 ± 0.11	3.27 ± 0.35
C22:1	Erucic acid	−	−	0.92 ± 0.05	1.70 ± 0.10	11.20 ± 1.09	2.34 ± 0.12	2.39 ± 0.11	2.14 ± 0.08	−
C24:0	Lignoceric acid	−	−	3.73 ± 1.12	3.19 ± 1.17	−	9.53 ± 1.95	8.40 ± 2.01	11.33 ± 2.21	−
C24:1	Nervonic acid	−	−	−	−	−	2.40 ± 0.09	2.17 ± 0.11	1.82 ± 0.07	−
C20:5	Eicosapentaenoic acid	−	−	−	−	8.00 ± 1.43	6.26 ± 1.65	8.30 ± 1.72	7.26 ± 1.56	−
C22:6	Docosahexaenoic acid	−	−	−	−	10.60 ± 2.11	7.11 ± 1.68	9.78 ± 1.76	9.97 ± 1.83	−
Other		−	0.90 ± 0.24	1.80 ± 0.09	1.33 ± 0.08	2.00 ± 0.11	1.38 ± 0.12	2.08 ± 0.17	2.79 ± 0.13	1,6 ± 0.09
SFA (saturated fatty acids)		20.66 ± 0.31	12.40 ± 0.27	36.25 ± 0.39	30.18 ± 0.52	23.40 ± 0.47	32.06 ± 1.12	26.89 ± 0.82	30.17 ± 1.17	16.51 ± 0.29
MUFA (monounsaturated fatty acids)		77.12 ± 1.20	76.10 ± 1.87	52.38 ± 1.92	59.03 ± 1.89	50.00 ± 1.82	44.18 ± 1.79	43.08 ± 1.59	42.23 ± 1.45	63.45 ± 0.60
PUFA (polyunsaturated fatty acids)		2.22 ± 0.47	10.60 ± 0.51	9.57 ± 0.60	9.46 ± 0.87	24.60 ± 1.84	22.38 ± 1.76	27.95 ± 1.69	24.81 ± 1.75	18.44 ± 1.02

**Table 3:** Parameters of storage lipids biosynthesis in a batch bioreactor culture of *Y. lipolytica* in media with fish waste oil as a sole carbon source (MF5) and MO5 (olive oil) and MG7 (glucose) control media. The variants with oxygenation (MF5-O_2_) and pH regulation (MF5-pH) were also included.

Parameter	Unit	Medium				
		MG7	MO5	MF5	MF5-O_2_	MF5-pH
Initial concentration of carbon source [S]	g/dm^3^	70	50	50	50	50
Time [t]	h	40	38	70	94	119
Duration of lag phase [t_lag_]	h	17	13	37	47	49
Biomass yield [X]	g d.m./dm^3^	13.79	23.10	15.16	20.43	8.09
Maximum concentration of lipids produced [L_max_]	g/dm^3^	1.15	7.88	1.43	4.64	1.90
Conversion yield of biomass per carbon substrate [Y_X/S_]	g d.m./g	0.28	0.46	0.30	0.41	0.16
Conversion yield of storage lipids per biomass formed [Y_L/X_]	g/g d.m.	0.080	0.340	0.094	0.227	0.234
Conversion yield of storage lipids per carbon substrate [Y_L/S_]	g/g	0.020	0.160	0.029	0.093	0.038
Volumetric rate of storage lipids production [q_Lv_]	g/dm^3^/h	0.030	0.210	0.020	0.049	0.016
Specific rate of storage lipid production [q_L_]	g/g d.m./h	0.0020	0.0090	0.0010	0.0024	0.0020

## Supplementary Materials

The following are available online at https://www.mdpi.com/2304-8158/10/2/436/s1, Table S1: Profile of fatty acids in carbon source (waste fish oil) and microbial oil extracted from yeast cells grown in MF5 medium. During batch cultures, some modifications of culture conditions were implemented (oxygenation MF5-O_2_; pH regulation MF5-pH) [content of fatty acids in relations to total fatty acids concentration, %], average standard deviation ±1.50.; Table S2: Extrapolated PDSC thermooxidation onset temperatures (t_ON_/°C) measured at different heating rates (*β*) for microbial oils extracted from yeast cells cultured in waste fish oil (MF5) and olive oil medium (MO5). Table S3: Kinetic parameters characterizing the thermooxidation of microbial oils extracted from yeast cells cultured in waste fish oil (MF5) and olive oil medium (MO5). Table S4: Total phenolic content in microbial oils extracted from yeast cells cultured in waste fish oil (MF5) and olive oil medium (MO5) expressed as milligram gallic acid per gram of oil (mg GA/g oil).
