# Supplementary material for: Studies on Upgradation of Waste Fish Oil to Lipid-Rich Yeast Biomass in Yarrowia lipolytica Batch Cultures
Source: Foods. 2021 Feb 17;10(2):436. doi: 10.3390/foods10020436 (PMC7922964; doi:10.3390/foods10020436)
Supplement: Supplementary file 1 [file foods-10-00436-s001.pdf]

foods MDPI

Upgradation of Waste Fish Oil to Single Cell Oil by *Yarrowia lipolytica* Yeast Cells

Agata Urszula Fabiszewska [1,*], Bartłomiej Zieniuk [1], Mariola Kozłowska [1], Patrycja Maria Mazurczak-Zieniuk [1], Małgorzata Wołoszynowska [2], Paulina Misiukiewicz-Stępień [3] and Dorota Nowak [4]

[1] Department of Chemistry, Institute of Food Sciences, Warsaw University of Life Sciences-SGGW, 159c Nowoursynowska Street, 02-776 Warsaw, Poland; bartlomiej_zieniuk@sggw.edu.pl (B.Z.); mariola_kozlowska@sggw.edu.pl (M.K.); patrycja.mazurczak@op.pl (P.M.M.-Z.)
[2] Łukasiewicz Research Network—Institute of Industrial Organic Chemistry, 6 Annopol Street, 03-236 Warsaw, Poland; malgorzata.woloszynowska@ipo.lukasiewicz.gov.pl
[3] Postgraduate School of Molecular Medicine, Medical University of Warsaw, 2a Trojdena Street, 02-091 Warsaw, Poland; pmisiukiewicz@wum.edu.pl
[4] Department of Food Engineering and Process Management, Institute of Food Sciences, Warsaw University of Life Sciences-SGGW, Nowoursynowska Street 159c, 02-776 Warsaw, Poland; dorota_nowak@sggw.edu.pl
* Correspondence: agata_fabiszewska@sggw.edu.pl; Tel.: +48-22-59-37-621

Table 1. Profile of fatty acids in carbon source (waste fish oil) and microbial oil extracted from yeast cells grown in MF5 medium, during batch cultures some modifications of culture conditions were implemented (oxygenation MF5-O_2; pH regulation MF5-pH) [content of fatty acids in relations to total fatty acids concentration, %], average standard deviation ± 1.50.

Fatty acid		Waste fish oil	Microbial oil from *Y. lipolytica* cells grown in MF5-O_2 medium			Microbial oil from *Y. lipolytica* cells grown in MF5-pH medium
			46 h	72 h	96 h	96 h
**Symbol**	**Name**					
C14:0	Myristic acid	8.10 ± 0.29	1.19 ± 0.14	4.80 ± 0.52	9.39 ± 0.25	3.48 ± 0.18
C16:0	Palmitic acid	12.10 ± 0.34	14.42 ± 0.25	12.70 ± 0.26	9.32 ± 0.15	14.94 ± 0.52
C16:1	Palmitoleic acid	11.50 ± 0.78	8.79 ± 0.56	10.34 ± 0.68	11.49 ± 0.84	5.30 ± 0.39
C18:0	Stearic acid	3.20 ± 0.15	3.93 ± 0.21	2.76 ± 0.09	5.34 ± 0.16	3.10 ± 0.20
C18:1	Oleic acid	17.30 ± 2.03	33.86 ± 1.22	31.39 ± 1.75	18.55 ± 1.86	25.72 ± 1.06
C18:2	Linoleic acid	1.40 ± 0.76	6.17 ± 0.50	5.97 ± 0.87	17.17 ± 0.62	1.49 ± 0.13
C18:3	Linolenic acid	4.60 ± 0.21	0.03 ± 0.01	0.39 ± 0.07	0.07 ± 0.02	1.07 ± 0.12
C20:0	Arachidic acid	-	-	-	-	3.16 ± 0.19
C20:1	Eicosenic acid	10.00 ± 1.67	2.42 ± 0.53	6.28 ± 0.25	1.18 ± 0.12	10.54 ± 1.08
C22:0	Behenic acid	-	-	-	-	-
C22:1	Erucic acid	11.20 ± 1.09	12.88 ± 0.98	5.61 ± 0.69	4.48 ± 0.84	11.05 ± 1.00
C24:0	Lignoceric acid	-	-	-	-	-
C24:1	Nervonic acid	-	2.20 ± 0.52	5.17 ± 0.40	12.23 ± 0.62	2.37 ± 0.36
C20:5	Eicosapentaenoic acid	8.00 ± 1.43	5.93 ± 1.14	8.06 ± 1.25	4.06 ± 0.57	7.60 ± 0.56
C22:6	Docosahexaenoic acid	10.60 ± 2.11	8.17 ± 1.35	6.53 ± 1.48	6.73 ± 1.65	10.17 ± 2.01
other		2.00 ± 0.11	-	-	-	-
SFA (saturated fatty acids)		23.40 ± 0.47	19.54 ± 0.20	20.26 ± 0.44	24.04 ± 0.28	24.04 ± 0.30

MUFA (monounsaturated fatty acids)	50.00 ± 1.82	60.15 ± 0.76	58.79 ± 0.75	47.93 ± 0.86	47.93 ± 0.78
PUFA (polyunsaturated fatty acids)	24.60 ± 1.84	20.31 ± 1.38	20.95 ± 1.37	28.02 ± 0.57	28.02 ± 1.30

Table 2. Extrapolated PDSC thermooxidation onset temperatures (t_{ON}/ºC) measured at different heating rates (β) for microbial oils extracted from yeast cells cultured in waste fish oil (MF5) and olive oil medium (MO5).

**Heating rate**	**Growth medium**	
**β/ºC min^{-1}**	**MF5**	**MO5**
4.0	124.29 ± 0.34	178.12 ± 0.78
7.5	133.39 ± 0.47	186.96 ± 0.93
10.0	137.53 ± 0.49	192.94 ± 1.04
12.5	141.26 ± 0.53	198.78 ± 1.13
15.0	144.22 ± 0.47	202.38 ± 1.39

Table 3. Kinetic parameters characterizing the thermooxidation of microbial oils extracted from yeast cells cultured in waste fish oil (MF5) and olive oil medium (MO5).

**Parameters**	**Growth medium**	
	**MF5**	**MO5**
-a and b	4.790 and 12.66	4.961 and 11.62
r^2	0.999	0.986
E_a (kJ mol^{-1})	87.21	90.33
logZ	10,95	9.90
τ at 140 °C (min)	1.19	33.33
τ at 150 °C (min)	0.65	17.86

Table 4. Total phenolic content in microbial oils extracted from yeast cells cultured in waste fish oil (MF5) and olive oil medium (MO5) expressed as milligram gallic acid per gram of oil (mg GA/g oil).

**Growth medium**	**Concentration of phenolic compounds in microbial oil [mg gallic acid/g oil]**
MF5	1.22 $^{\pm 0.001}$
MO5	0.035 $^{\pm 0.0001}$
